# Immunotherapy‐Induced Type 1 Diabetes Mellitus Causing Diabetic Ketoacidosis: A Case Report and Review of Current Guidelines

**DOI:** 10.1002/cnr2.70058

**Published:** 2024-11-19

**Authors:** Patrick Disterhaft, Caleb Kerr, Daniel Barnett, Moti Salloum

**Affiliations:** ^1^ Kirksville College of Osteopathic Medicine A.T. Still University Kirksville USA; ^2^ Internal Medicine Residency Program University of Arizona College of Medicine ‐ Phoenix Phoenix USA; ^3^ Phoenix VA Medical Center Phoenix USA; ^4^ University of Arizona College of Medicine ‐ Phoenix Phoenix USA

**Keywords:** diabetic ketoacidosis, immune checkpoint inhibitors, ipilimumab, malignant mesothelioma, nivolumab, type 1 diabetes mellitus

## Abstract

**Background:**

Immune checkpoint inhibitors (ICIs) are becoming more frequently used in the treatment of many types of malignant cancers by disinhibiting T‐cell activation, which promotes the destruction of cancer cells. This disinhibition can also result in autoimmune conditions, like endocrinopathies.

**Case:**

We report a case of a 78‐year‐old male patient with malignant mesothelioma treated with combination ICI therapy who presented with diabetic ketoacidosis (DKA) with no history of diabetes mellitus or hyperglycemia. The patient was admitted to the intensive care unit and treated with intravenous (IV) fluid repletion and IV insulin for DKA. The patient was diagnosed with new‐onset type 1 diabetes mellitus (T1DM) induced by ICI therapy.

**Discussion:**

Approximately 75% of patients diagnosed with ICI‐induced T1DM initially present with DKA. This, along with the rapid onset of hyperglycemia in this patient, suggests current guidelines for monitoring blood glucose are inadequate. Current guidelines recommend monitoring blood glucose at the following times: baseline, at the initiation of each cycle for 12 weeks, and then every 3–6 weeks thereafter. We propose the following schedule for monitoring blood glucose in patients receiving ICI therapy: baseline, twice weekly for the first six cycles, and then once weekly thereafter. This proposed update is supported by our patient's rapid onset of hyperglycemia and other case reports and reviews showing that most patients with this diagnosis have an initial presentation of DKA. Detecting hyperglycemia and starting treatment early is important in the prevention of acute complications from uncontrolled T1DM, like DKA.

**Conclusion:**

This case adds to the existing body of literature and provides support for more frequent monitoring of blood glucose in patients receiving ICI therapy. Blood glucose monitoring is a simple, reliable, low risk, and inexpensive laboratory test that should be used in patients receiving ICI therapy to ensure prompt diagnosis and treatment of T1DM.

## 1 Introduction

**FIGURE 1:**
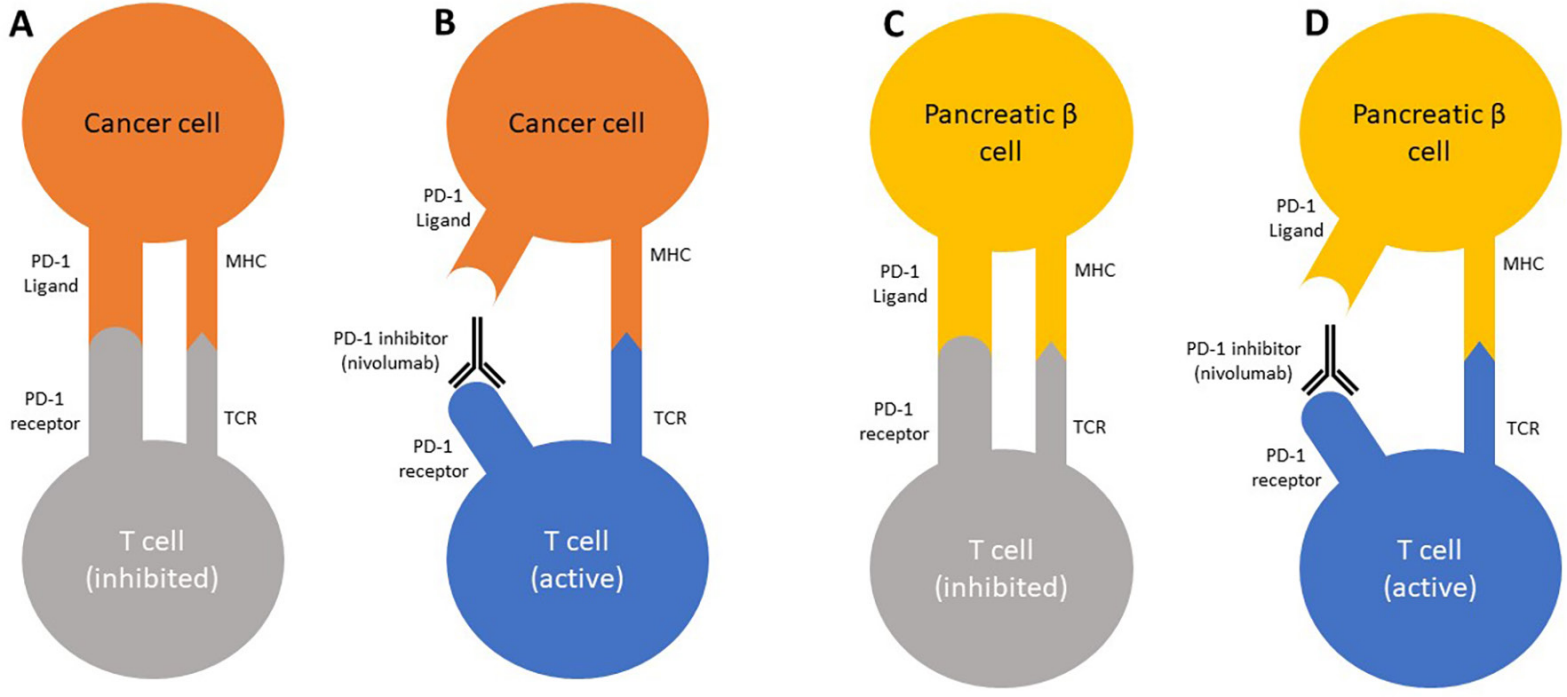
Mechanism of action of PD‐1 inhibitors. (A) Cancer cells upregulate PD‐1 ligand, which causes the inhibition of T cells and allows the cancer cell to survive. (B) PD‐1 inhibitors block the binding of PD‐1 and PD‐1 ligand, which causes the activation of T cells against the cancer cell. (C) Normal, healthy pancreatic β cells express PD‐1 ligand and bind to PD‐1 on T cells to prevent autoimmunity. (D) PD‐1 inhibitors block the binding of PD‐1 and PD‐1 ligand, which causes activation of T cells against normal, healthy pancreatic β cells. Figure 1 was redrawn based on a figure from Clotman and colleagues in 2018 [11].

Immune checkpoint inhibitors (ICIs) have become increasingly common therapy for the treatment of malignant cancers, having improved the prognosis for patients with many types of malignant cancers [1]. Immune checkpoint receptors, like programmed death receptor 1 (PD‐1) and cytotoxic T‐lymphocyte associated antigen 4 (CTLA‐4) are found on T cells and play a role in the regulation of immune responses and the prevention of autoimmunity [2]. PD‐1 and CTLA‐4 bind to immune checkpoint proteins displayed by healthy cells and inhibit T cell‐mediated destruction of those cells. Many cancer cells upregulate and display these immune checkpoint proteins, which downregulates immune responses toward them. ICIs are monoclonal antibodies that work by binding to the PD‐1 and CTLA‐4 receptors on T cells, thus inhibiting their inhibitory effect. This disinhibition allows T cells to target and destroy cancer cells (Figure 1). Unfortunately, this disinhibition can also result in autoantibody development and subsequent autoimmune‐related diseases. A significant percentage of patients treated with ICIs develop endocrinopathies, including hypophysitis, thyroid dysfunction, type 1 diabetes mellitus (T1DM), and adrenal insufficiency [3]. T1DM is considered an uncommon to common adverse effect of ICI therapy. In clinical trials, the reported incidence of T1DM was 0.9% with nivolumab monotherapy and 2.7% with nivolumab/ipilimumab combination therapy, with no reported incidences of T1DM with ipilimumab monotherapy [4, 5]. Several case reports and systematic reviews have been published showing the association between ICI therapy and DKA caused by new‐onset T1DM [6, 7, 8, 9, 10, 11]. In clinical trials, the reported range for onset of type 1 diabetes in nivolumab monotherapy was 15 days to 21.9 months, with the median being 4.4 months [4]. Depending on the dosage, the reported range for nivolumab/ipilimumab combination therapy was 19 days to 16.8 months, with the median being 2.5–3.3 months [4]. Two systematic reviews by Lin and colleagues and by de Filette and colleagues found that in clinical practice, the average number of cycles of PD‐1/CTLA‐4 inhibitor combination therapy at the time of T1DM diagnosis is 6.11 cycles (range 1–28 cycles) and 2.7 cycles (range 1–5 cycles), respectively [8, 12]. In clinical practice, it has also been shown that T1DM is far more common with PD‐1 inhibitor monotherapy and PD‐1/CTLA‐4 inhibitor combination therapy than CTLA‐4 inhibitors only, though de Filette and colleagues do report three cases of T1DM caused by CTLA‐4 inhibitor monotherapy [8, 12]. In addition, those same reviews found that around 75% of ICI‐induced T1DM cases initially present as diabetic ketoacidosis (DKA) [8, 12]. We report a case of DKA secondary to new‐onset T1DM in a patient with malignant mesothelioma being treated with nivolumab, a PD‐1 inhibitor, and ipilimumab, a CTLA‐4 inhibitor.

## 2 Case Report

### 2.1 Case Presentation

A 78‐year‐old male patient with a past medical history of unresectable malignant mesothelioma, hypertension, hyperlipidemia, atrial fibrillation, and benign prostatic hyperplasia presented to the emergency department in Phoenix, AZ in January 2024 with a 2‐week history of polydipsia, polyuria, and fatigue. The patient also endorsed nausea, xerostomia, and weight loss. He denied headache, dizziness, shortness of breath, chest pain, palpitations, vomiting, abdominal pain, diarrhea, constipation, hematochezia, or melena. Past surgical history includes partial colectomy, cholecystectomy, and abdominal hernia repair. The patient's current medications include atorvastatin, apixaban, aspirin, metoprolol, and tamsulosin. The patient is also on combination immunotherapy with nivolumab and ipilimumab and completed his fourth cycle of therapy 16 days prior to admission. He began immunotherapy in October 2023 with an infusion schedule of nivolumab every 3 weeks and ipilimumab every 6 weeks. The patient has a remote 5 pack‐year history of smoking, drinks one glass of wine weekly, and denies any illicit drug use.

The patient's vital signs were within normal limits and stable. On physical exam, he appeared tired, mucous membranes were dry, and skin turgor was increased. Other physical exam findings were unremarkable. The patient was alert and oriented without evidence of acute distress. The lungs were clear to auscultation bilaterally without crackles, rales, or wheezes. The heart had a regular rate and rhythm on auscultation without any obvious murmurs, rubs, or gallops. The abdomen had normoactive bowel sounds; there was no abdominal erythema, bruising, distention, tenderness, or guarding.

Initial laboratory investigation (Table 1) revealed hyperglycemia (757 mg/dL), ketonemia (6.31 mmol/L), ketonuria (20 mg/dL), glucosuria (> 500 mg/dL), metabolic acidosis (bicarbonate 17 mmol/L), elevated anion gap (25.0 mEq/L), and acute kidney injury (creatinine 2.00 mg/dL).

**TABLE 1:** Admission laboratory results.

	Result	Reference range
Complete blood count		
White blood cell (K/μL)	8.3	3.5–10.6
Red blood cell (M/μL)	5.46	4.40–5.90
Hemoglobin (g/dL)	15.9	13.0–18.0
Hematocrit (%)	47.3	40.0–52.0
Mean corpuscular volume (fL)	86.6	80.0–100.0
Platelets (K/μL)	215	150–440
Electrolyte profile		
Sodium (mmol/L)	142	135–145
Potassium (mmol/L)	5.1 (H)	3.5–5.0
Chloride (mmol/L)	100	98–110
Bicarbonate (mmol/L)	17 (L)	18–29
Magnesium (mg/dL)	2.8 (H)	1.6–2.5
Phosphate (mg/dL)	6.2 (H)	2.7–4.5
Liver profile		
Aspartate transaminase (U/L)	28	10–40
Alanine transaminase (U/L)	36	10–35
Alkaline phosphatase (U/L)	142 (H)	53–128
Bilirubin, total (mg/dL)	2.0 (H)	0.2–1.0
Protein, total (g/dL)	8.9 (H)	6.4–8.5
Albumin (g/dL)	4.8	3.5–5.1
Renal profile		
Blood urea nitrogen (mg/dL)	42 (H)	7–20
Creatinine (mg/dL)	2.00 (H)	0.72–1.25
Other laboratory results		
Anion gap (mEq/L)	25.0 (H)	9.0–18.0
Glucose (mg/dL)	757 (H)	70–100
Hemoglobin A1C (%)	10.1 (H)	4.4–6.4
Serum ketones (mmol/L)	6.31 (H)	0.00–0.30
Urinalysis		
Specific gravity	1.031	1.005–1.050
pH	6.0	5.0–8.0
Protein (mg/dL)	30 (H)	Neg
Glucose (mg/dL)	> 500 (H)	Neg
Ketones (mg/dL)	20 (H)	Neg
Blood	Neg	Neg
White blood cells	1/HPF	0–5
Red blood cells	2/HPF	0–3
Hyaline casts	6–10/LPF (H)	0–2
Bacteria	Neg	Neg
Leukocyte esterase	Neg	Neg
Nitrite	Neg	Neg

### 2.2 Differential Diagnosis and Initial Plan

Given the presenting symptoms (polydipsia, polyuria, fatigue, and nausea) and laboratory results (severe hyperglycemia, ketonemia, and elevated anion gap metabolic acidosis) were consistent with a classic presentation of DKA, our most likely diagnosis was DKA. The remainder of our differential diagnosis included hyperosmolar hyperglycemic state, alcoholic ketoacidosis, undiagnosed type 2 diabetes mellitus, and toxic ingestion of methanol, ethylene glycol, or salicylate. Since the patient denied alcohol overuse, denied any chance or situation of ingesting any toxic substance, and had previously normal fasting blood glucose levels, hyperglycemia secondary to another cause was suspected.

The patient was admitted to the intensive care unit (ICU) and treatment for DKA was initiated: 1 L/h of normal saline and intravenous (IV) insulin bolus of 0.1 units/kg followed by continuous insulin infusion of 0.1 units/kg/h. Electrolytes and blood glucose were monitored every 2 h.

### 2.3 Final Diagnosis, Treatment, and Follow‐Up

Chart review and patient history revealed no previous diagnosis of diabetes mellitus or any history of hyperglycemia. Previous fasting blood glucose ranged from 90 to 110 mg/dL, with the most recent fasting blood glucose result being 104 mg/dL 37 days prior to hospitalization. Hemoglobin A1C (HbA1C) during admission was 10.1%. The patient responded appropriately to treatment and was transitioned to subcutaneous insulin on day two following the resolution of ketoacidosis. After laboratory results returned to within reference ranges, the patient's care was transferred to the medicine service on day 3.

The patient was diagnosed with DKA secondary to ICI‐induced T1DM. The patient was discharged home on multiple daily injections of insulin, with a total daily dose of 1.2 units/kg/day. The patient was counseled on proper diabetes management and scheduled for an outpatient endocrinology consultation 4 days after discharge. Following that consultation, the patient was prescribed a continuous glucose monitor. His insulin regimen was adjusted to a total daily dose of 0.5 units/kg/day, and he will continue to ensure strict glycemic control. After achieving control of his blood glucose level, the patient received his fifth cycle of immunotherapy 23 days after hospitalization. At 65 days following discharge from the hospital, the patient's HbA1C was 7.9%. The patient continues to be compliant with his follow‐up appointments and his insulin regimen at the time of writing this manuscript.

**FIGURE 2:**
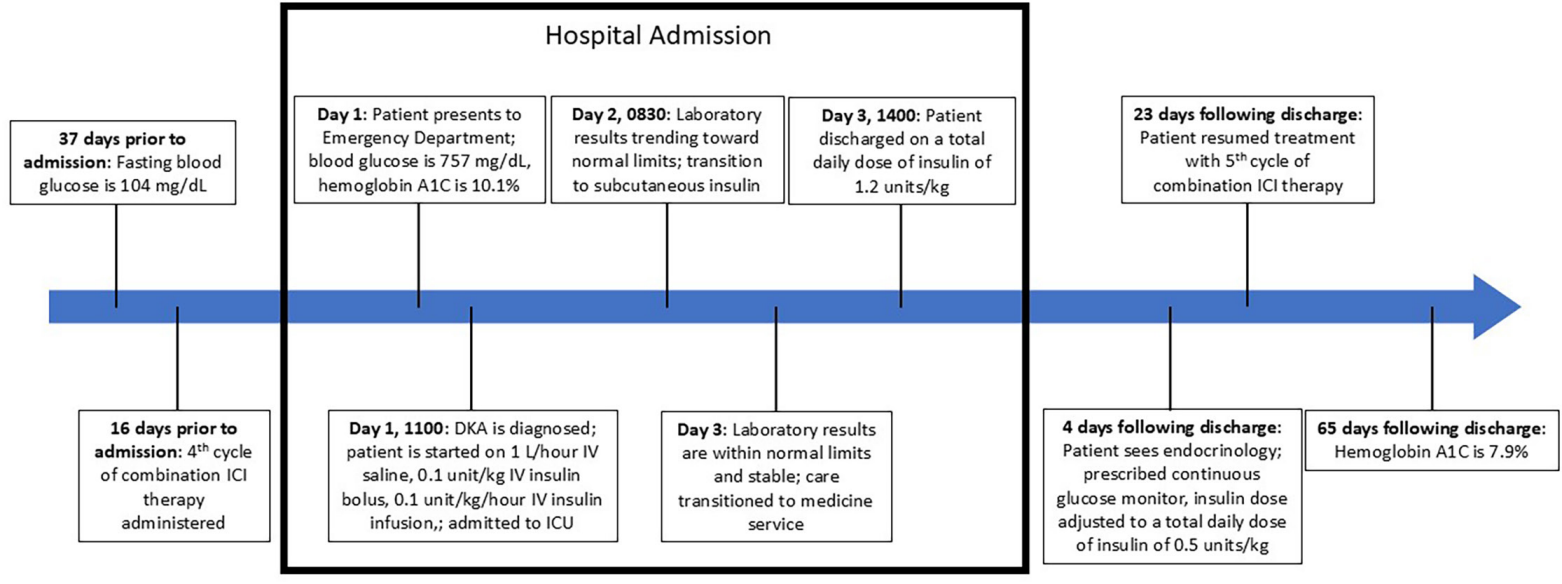
A timeline of the patient's clinical course. Hospital events are shown within the black box. Pre‐ and post‐hospital events are shown outside the black box.

Subsequent laboratory testing (Table 2) showed low serum C‐peptide, negative glutamic acid decarboxylase antibodies (GADA), and negative islet cell antibodies (ICA). An abbreviated version of the patient's clinical course can be seen in Figure 2.

**TABLE 2:** Autoimmune diabetes panel.

	Result	Reference range
GADA^a^ (U/mL)	< 5	< 5
ICA^b^	Neg	Neg
C‐peptide (ng/mL)	0.67 (L)	0.8–3.85

^a^ Glutamic acid decarboxylase antibodies.

^b^ Islet cell antibodies.

## 3 Discussion

As shown in Figure 1, the binding of the PD‐1 ligand on a host cell to the PD‐1 receptor on a T cell prevents autoimmune targeting by inhibiting the T cell. Although both CTLA‐4 and PD‐1 play important roles in autoimmunity, PD‐1 has been shown to play an especially important role in the development of autoimmune T1DM by inhibiting the expansion of autoreactive T cells [2, 13]. This is supported by the fact that T1DM is an adverse effect associated with PD‐1 inhibitors, like nivolumab, while the use of CTLA‐4 inhibitor ipilimumab as monotherapy was not associated with T1DM in clinical trials [3, 4]. However, the addition of ipilimumab to nivolumab for combination therapy appears to increase the incidence of T1DM compared to nivolumab only [4]. In addition to combination therapy, other risk factors in developing ICI‐induced T1DM are young age (age ≤ 60) and pre‐existing non‐type 1 diabetes mellitus [14].

Diagnosis of ICI‐induced T1DM is similar to traditional diabetes diagnosis: persistently elevated fasting blood glucose and/or elevated HbA1C. As with traditional T1DM, the presence of certain autoantibodies can be useful in diagnosis. ICA, GADA, and insulin antibodies (IAA) as well as C‐peptide levels are typical laboratory tests for T1DM. Based on our patient and other recent case reports, the presence of autoantibodies alone is not sufficient for diagnosis. Several analyses have shown that autoantibody laboratory tests are positive in 33%–53% of patients with ICI‐induced T1DM [8, 12, 15]. Although the presence of autoantibodies can be useful to support a diagnosis of ICI‐induced T1DM, their absence should not rule it out.

Although glucocorticoids may be used to manage and reverse most other ICI‐induced endocrinopathies, patients with ICI‐induced T1DM should not be treated with glucocorticoids as this may exacerbate hyperglycemia in these patients [3, 16]. The damage to pancreatic beta cells is irreversible, thus glucocorticoid treatment will not help slow or reverse the disease process [3, 16]. The current treatment recommendation for ICI‐induced T1DM is insulin therapy, similar to traditional T1DM [16, 17]. In patients who present with DKA, ICI therapy should be stopped while the patient undergoes treatment for DKA. ICI therapy may be resumed following the resolution of DKA and hyperglycemia [16, 17].

Since hyperglycemia seems to be the only reliable indicator of ICI‐induced T1DM, fasting blood glucose and HbA1C should be checked prior to the initiation of ICI therapy and monitored frequently throughout therapy to ensure prompt detection of hyperglycemia. The current guidelines from the American Society of Clinical Oncology recommend monitoring blood glucose at baseline, at the beginning of each cycle for 12 weeks, and every 3–6 weeks thereafter [17]. In patients diagnosed with ICI‐induced T1DM, 71%–76% have an initial presentation of DKA [8, 12]. This suggests that patients on ICI therapy are not being adequately monitored for hyperglycemia, which is resulting in costly, preventable hospital admissions in an already strained healthcare system. With the average time to diagnosis generally being within the first six cycles, this timeframe seems to be most important in monitoring for hyperglycemia. Based on these findings as well as our patient's history and presentation, we propose the following schedule for monitoring blood glucose: at baseline, twice weekly for the first six cycles, and then once weekly thereafter. This schedule allows for close monitoring of blood glucose through the median time to onset of ICI‐induced T1DM and then continued, less stringent monitoring thereafter to identify later onset cases. Although we recommend this glucose monitoring for every patient on ICI therapy, it may be appropriate to adopt a modified, less stringent schedule for patients on CTLA‐4 inhibitor monotherapy. There are reports of T1DM with CTLA‐4 inhibitor use, but the incidence appears to be far lower than in PD‐1 inhibitor monotherapy or PD‐1/CTLA‐4 inhibitor combination therapy [8]. In addition to laboratory testing, patients and their family members should be educated on the signs and symptoms of hyperglycemia and DKA, including polydipsia, polyuria, weight loss, lethargy, nausea, vomiting, abdominal pain, and altered mental status, to ensure prompt recognition outside the clinical setting. Along with our blood glucose monitoring recommendation, a clinician may consider prescribing a glucose monitor for patients who are willing and able to monitor their blood glucose at home. This could help minimize the travel burden for the patient and reduce excessive laboratory visits as a result of this new recommendation.

## 4 Conclusion

New‐onset T1DM is a serious adverse effect of ICI therapy that often initially presents as DKA. This paper adds to the existing body of literature showing the association between T1DM and ICI therapy. In addition, we believe an update to the guidelines is warranted to reflect the current evidence that suggests inadequate blood glucose monitoring in these patients. Monitoring blood glucose at baseline, twice weekly for the first six cycles, and then once weekly thereafter is a reasonable recommendation and one that could prevent complications secondary to undiagnosed T1DM. Blood glucose monitoring is a simple, reliable, inexpensive, and low‐risk laboratory test that is essential for all patients on ICI therapy given the rapid onset and detrimental outcomes associated with uncontrolled T1DM. Detecting hyperglycemia early ensures prompt diagnosis of T1DM and allows early treatment initiation before the development of acute complications of uncontrolled T1DM, like DKA, in patients receiving ICI therapy.

## Author Contributions

**Patrick Disterhaft:** conceptualization, writing – original draft, writing – review and editing. **Caleb Kerr:** conceptualization, writing – review and editing. **Daniel Barnett:** conceptualization, writing – review and editing. **Moti Salloum:** conceptualization, writing – review and editing, supervision.

## Ethics Statement

Ethical approval from IRB is not applicable to this article. This article does not contain any studies with human or animal subjects. Written informed consent was obtained from the patient for their anonymized information to be published in this article.

## Conflicts of Interest

The authors declare no conflicts of interest.

## Data Availability

Data sharing is not applicable to this article as no new data were created or analyzed in this study.
